# Adaptive Robust Unscented Kalman Filter via Fading Factor and Maximum Correntropy Criterion

**DOI:** 10.3390/s18082406

**Published:** 2018-07-24

**Authors:** Zhihong Deng, Lijian Yin, Baoyu Huo, Yuanqing Xia

**Affiliations:** 1 The School of Automation, Beijing Institute of Technology, Beijing 100081, China; 20140378@bit.edu.cn (L.Y.); xia_yuanqing@bit.edu.cn (Y.X.); 2 The Second Construction Limited Company of China Construction Eighth Engineering Division, Jinan 250014, China; 3120130379@bit.edu.cn

**Keywords:** maximum correntropy criterion, tracking target, unscented transform, adaptive robust control

## Abstract

In most practical applications, the tracking process needs to update the data constantly. However, outliers may occur frequently in the process of sensors’ data collection and sending, which affects the performance of the system state estimate. In order to suppress the impact of observation outliers in the process of target tracking, a novel filtering algorithm, namely a robust adaptive unscented Kalman filter, is proposed. The cost function of the proposed filtering algorithm is derived based on fading factor and maximum correntropy criterion. In this paper, the derivations of cost function and fading factor are given in detail, which enables the proposed algorithm to be robust. Finally, the simulation results show that the presented algorithm has good performance, and it improves the robustness of a general unscented Kalman filter and solves the problem of outliers in system.

## 1. Introduction

In real-world applications, target tracking problems have attracted much attention such as maneuvering target tracking [1], ballistic target tracking [2] and multiple target tracking [3], etc. For getting better accuracy, efficiency and performance of tracking problems, the effect of noise needs to be reduced, especially of measurement noise. Therefore, the measurement noise needs to be restrained timely in the process of a tracking target. As is well known, filtering algorithms are powerful tools for suppressing the effect of noise. Kalman [4] first proposed the Kalman filter (KF) algorithm in 1960, and the filtering technique has developed quickly ever since. For the linear Gaussian process [4,5,6,7], the KF could get optimal recursive results based on the minimum mean square error (MMSE) estimation. It is well known that KF can employ the first two moments (mean and covariance) of state and measurement to obtain optimal estimates. It can be seen that the KF has been applied in numerous areas such as navigation, target tracking, communications [7], attitude determination [8], multiple sensors data [9] and many more. However, for amounts of data collected or sent by sensors, the KF experienced heavy computational load to get an optimal solution. In most practical applications, the systems are nonlinear. In this case, the performance of KF is unsatisfactory. Furthermore, when a dynamic model was contaminated by outliers [10], the KF degraded severely. Therefore, it is necessary to design an efficient algorithm that can suppress the outliers for the nonlinear problem.

In fact, for a nonlinear tracking problem, many nonlinear filtering algorithms were proposed to deal with the nonlinear systems—for example, extended Kalman filter (EKF), unscented Kalman filter (UKF), cubature Kalman filter (CKF), particle filter (PF), Gaussian sum filter (GSF), and so on. Particularly, it is well known that EKF deals with the nonlinear system by its first-order Taylor Series expansions and leads to a suboptimal solution. UKF, based on unscented transform (UT), is an improved filter compared to KF and EKF. It selects deliberately as sigma points to generalize the KF for both linear and nonlinear systems, and it can be used to propagate three order information for state estimation. Therefore, it can achieve better estimation accuracy and computational feasibility [11]. Zhan and Wan [12] developed the iterated unscented Kalman filter for passive target tracking. Based on the spherical-radial cubature rule, CKF is presented to approximate the Gaussian filter [13,14]. GSFs are based on the idea that a non-Gaussian probability density function (PDF) can be approximated by a sum of Gaussian PDFs [15]. Based on Bayesian estimation and a sequential Monte Carlo approach, Du et al. utilized PF to handle nonlinear and non-Gaussian problems, and the PF is applied in small target tracking in an optimal image sequence [16]. However, these nonlinear filters were susceptible to outliers and did not have robust property.

For measurement model contaminated by outliers, several filtering algorithms have been proposed [17,18,19,20,21,22,23]. Based on the Huber function, Wang et al. [17] presented the derivative-free filter to manage the measurement outliers, but it did not suppress the state outliers well. For measurement outliers, Durovic and Kovacevic [18] utilized the M-estimation to deal with measurement outliers in the presence of disturbance uncertainty. It can not deal with both state and measurement outliers. Their performance will diverge if the state and measurement models were contaminated by outliers simultaneously. To solve a nonlinear system with heavy-tailed noise, Wang et al. [19] studied a robust information filter to solve the measurements with a large error from the estimation process. However, it did not embed the fading factor into the framework of information filter. Under this case, Karlgaard [20] proposed an adaptive robust nonlinear filtering algorithm to resist the effects of outliers. For both the state and measurement outliers, Gandhi and Mili [21] introduced a generalized maximum likelihood type KF. However, the KF was limited to a linear system, and the evaluation of Jacobian matrices in EKF could be nontrivial and this leads to degraded performance. Chang et al. [22] investigated a robust filter to suppress the state and measurement outliers, and it utilized the robust property of the Huber function. The $H_{\infty }$ filter has robustness to minimize the estimation error [23], and it can not accommodate the outliers well for outliers occurring randomly. However, these filters have robustness to some extent.

In order to enhance the robustness of the aforementioned filters, some fading factors were proposed to embed into the above filters to keep them stable and effective [24,25,26]. Wang et al. [24] introduced a modifiable fading factor to tackle the nonlinear estimation problem. After that, several researchers also investigated this problem. Yang et al. [25] investigated the adaptive robust filtering via the robust maximum likelihood estimation, but it can not control the dynamics model biases. Safarinejadian and Yousefi [26] proposed an adaptive fading memory KF to deal with static alignment of inertial navigation systems. Furthermore, Geng and Wang [27] utilized multiple fading factors in KF to handle the filtering divergence with inaccurate system noise. In [25,26,27], the fading factors were embedded into KF, but not nonlinear filters. Therefore, in order to overcome their shortcomings, Karlgaard [20] and Wang et al. [24] embedded the fading factors into nonlinear filters to handle nonlinear problems.

Motivated by the above discussion, this work proposes a new adaptive robust UKF scheme based on both fading factor and maximum correntropy criterion (MCC) to focus on the state estimation problems with measurement outliers. To the best knowledge of the authors, for the nonlinear tracking problem, the filtering algorithm based on MCC and fading factor had not been studied before. In our research, tracking a moving target by a sensor has been carried out to compare with UKF and an adaptive Huber unscented Kalman filter [24]. Furthermore, the prime dedications of this paper are as follows: (1) the cost function of the proposed filter is proposed, the fading factor is applied in the state model and the maximum correntropy criterion is used in the measurement model; (2) the proposed filter can suppress the effect of outliers effectively in the dynamics system (state or/and observation equation); and (3) the proposed filter is easy to perform and has better performance in the presence of outliers.

The structure of this paper is organized as follows. The Section 2 briefly introduces the fundamentals of the proposed filter. Section 3 proposes the adaptive robust unscented Kalman filter based on fading factor and MCC for a nonlinear system. Simulation results and comparisons are provided to confirm the feasibility and superiority of the proposed filter in Section 4. Finally, conclusions are shown in Section 5.

## 2. Fundamentals of the Proposed Filter

### 2.1. Maximum Correntropy Criterion

For any random variables *X* and *Y*, the correntropy is defined as

(1) $$V\left(X,Y\right)=E_{X,Y}\left[\rho \left(X,Y\right)\right]=\int \int \rho \left(x,y\right)dF_{X,Y}\left(x,y\right),$$

where E[·] represents the mean value, and ρ(·) is a real-valued continuous, symmetric and nonnegative definite kernel function, respectively. $F_{X,Y}\left(x,y\right)$ is the joint probability density function of *X* and *Y*. However, $F_{X,Y}\left(x,y\right)$ is usually unknown, and numbers of data samples, $(x_{i},y_{i}),$ (i=1,2,…,N) can be obtained. Therefore, the correntropy can be computed as follows:

(2) $$\tilde{V}\left(X,Y\right)=\frac{1}{N}\sum_{i=1}^{N}\rho \left(x_{i},y_{i}\right).$$

In theory, various kernel functions can be used. In general, the Gaussian kernel function is selected as follows:

(3) $$\kappa_{G}\left(e_{i}\right)=\kappa_{G}\left(x_{i}-y_{i}\right)=\frac{1}{\sqrt{2\pi \sigma }}\exp\left\{-\frac{e_{i}^{2}}{2\sigma^{2}}\right\},$$

where $e_{i}=x_{i}-y_{i}$, and σ is the kernel width of correntropy, respectively. If $e_{i}=0$, $\kappa_{G}\left(e_{i}\right)$ can reach its maximum value. Therefore, the cost function of MCC is expressed by

(4) $$J_{MCC}=\min\left(\sum_{i=1}^{N}\left(\kappa_{G}\left(0\right)-\kappa_{G}\left(e_{i}\right)\right)\right).$$

### 2.2. Cost Function of Adaptive Robust Kalman Filter

In this section, the cost function of the adaptive robust Kalman filter is derived by analyzing the following linear state model and measurement model:

(5) $$x_{k}=\Phi_{k|k-1}x_{k-1}+w_{k-1}\;\;\;\;\;y_{k}=H_{k}x_{k}+v_{k},$$

where *k* denotes discrete time, $x_{k}\in R^{n}$ represents n×1 system state vector at time *k*, $\Phi_{k|k-1}$ is the state transition matrix, $y_{k}\in R^{m}$ is m×1 system measurement vector at time *k*, $H_{k}$ is observation matrix, $w_{k-1}$ is the process noise with the covariance $Q_{k-1}$, $v_{k}$ is the measurement noise with the covariance $R_{k}$, and they are uncorrelated zero-mean Gaussian white noises.

For simplicity, the problem of state and measurement outliers is focused on in this work. For the linear state space model (5), a combined cost function is used to perform two different criterions to the state model and measurement model. Utilizing the Bayesian maximum likelihood, the posterior mean estimate is derived by minimizing the following function

(6) $${\hat{x}}_{k|k}=\arg\min(∥x_{k}-{\hat{x}}_{k|k-1}{∥}_{P_{k|k-1}^{-1}}^{2}+∥H_{k}x_{k}-y_{k}{∥}_{R_{k}^{-1}}^{2}),$$

where ${∥x∥}_{A}^{2}=x^{T}Ax$ is the quadratic form with respect to A (A is nonnegative definite matrix ); ${\hat{x}}_{k|k}$ is the posteriori estimate, ${\hat{x}}_{k|k-1}$ is the priori estimate, $P_{k|k-1}$ is the covariance matrix of ${\hat{x}}_{k|k-1}$.

Denoting $\xi_{k}=R_{k}^{-1/2}\left(H_{k}x_{k}-y_{k}\right)$ and utilizing MCC in Equation (4), the cost function of the filtering algorithm in Equation (6) is formulated as follows:

(7) $${\hat{x}}_{k|k}=\arg\min(∥x_{k}-{\hat{x}}_{k|k-1}{∥}_{P_{k|k-1}^{-1}}^{2}+\sum_{j=1}^{m}\left(k_{G}\left(0\right)-k_{G}\left(\xi_{k,j}\right)\right)),$$

where the term *m* is the dimension of the measurement model. Furthermore, the fading factor is embedded to strengthen the robustness property of the KF with model error. Then, the cost function in Equation (7) can be rearranged as

(8) $$\begin{array}{c} {\hat{x}}_{k|k}=\arg\min(∥x_{k}-{\hat{x}}_{k|k-1}{∥}_{{\left(\alpha_{k}P_{k|k-1}\right)}^{-1}}^{2}+\sum_{j=1}^{m}\left(k_{G}\left(0\right)-k_{G}\left(\xi_{k,j}\right)\right)). \end{array}$$

Differencing Equation (8) with respect to $x_{k}$, one has that

(9) $${\left(\alpha_{k}P_{k|k-1}\right)}^{-1}\left(x_{k}-{\hat{x}}_{k|k-1}\right)-\sum_{i=1}^{n}\frac{\partial k_{G}\left(\xi_{k,i}\right)}{\partial \xi_{k,i}}\frac{\partial \xi_{k,i}}{\partial x_{k}}=0.$$

Substituting Equation (3) into Equation (9), it can be obtained that

(10) $${\left(\alpha_{k}P_{k|k-1}\right)}^{-1}\left(x_{k}-{\hat{x}}_{k|k-1}\right)+\frac{1}{\sqrt{2\pi }\sigma^{3}}\sum_{i=1}^{m}\exp\left(-\frac{\xi_{k,i}^{2}}{2\sigma^{2}}\right)\xi_{k,i}\frac{\partial \xi_{k,i}}{\partial x_{k}}=0.$$

Define the function

(11) $$\Psi_{j}=\frac{1}{\sqrt{2\pi }\sigma^{3}}\sum_{i=1}^{m}\exp\left(-\frac{\xi_{k,i}^{2}}{2\sigma^{2}}\right)$$

and the matrix

(12) $$\Psi =\mathrm{diag}[\Psi_{j}]\;\;\;j=1,\ldots ,m.$$

Thus, we find out that Equation (10) can be redescribed as follows:

(13) $${\left(\alpha_{k}P_{k|k-1}\right)}^{-1}\left(x_{k}-{\hat{x}}_{k|k-1}\right)+H_{k}^{T}R^{-T/2}\Psi \xi_{k}=0.$$

Substituting $\xi_{k}$ into Equation (13), we arrive at

(14) $${\left(\alpha_{k}P_{k|k-1}\right)}^{-1}\left(x_{k}-{\hat{x}}_{k|k-1}\right)+H_{k}^{T}R^{-T/2}\Psi R_{k}^{-1/2}\left(H_{k}x_{k}-y_{k}\right)=0.$$

Equation (14) satisfies the minimization solution of the cost function as follows:

(15) $${\hat{x}}_{k|k}=\arg\min(∥x_{k}-{\hat{x}}_{k|k-1}{∥}_{{\tilde{P}}_{k|k-1}^{-1}}^{2}+∥H_{k}x_{k}-y_{k}{∥}_{{\tilde{R}}_{k}^{-1}}^{2}),$$

where ${\tilde{P}}_{k|k-1}=\alpha_{k}P_{k|k-1}$ and ${\tilde{R}}_{k}=R_{k}^{T/2}\Psi_{k}^{-1}R_{k}^{1/2}$. Comparing Equation (15) with Equation (6), it can be seen that covariance matrixes of them are different, and others are identical. According to the iterative equations of KF, the explicit solution of Equation (15) can be performed as

(16) $$K_{k}={\tilde{P}}_{k|k-1}H_{k}^{T}{\left(H_{k}{\tilde{P}}_{k|k-1}H_{k}^{T}+{\tilde{R}}_{k}\right)}^{-1},$$

(17) $${\hat{x}}_{k|k}={\hat{x}}_{k|k-1}+K_{k}\left(y_{k}-H_{k}{\hat{x}}_{k|k-1}\right),$$

(18) $${\tilde{P}}_{k|k}=\left(I_{n}-K_{k}H_{k}\right){\tilde{P}}_{k|k-1}.$$

**Remark** **1.** *From an engineering viewpoint, it can be seen that the real state vector $x_{k}$ can not be obtained. However, some methods are proposed to change the proportion of state vector, and they can not utilize the state vector—for example, maximum correntropy criterion, Huber function, and so on. Although the state vector was coped with in [22], the real state $x_{k}$ was replaced by estimation vector ${\hat{x}}_{k|k-1}$ in the process.*

### 2.3. Formation of the Fading Factor

The state estimate of Kalman filter depends on the ratio of new measurements and the ones which are based on predicted state vector, dynamics model, and all previous measurements. If the state model error is much larger than that of the measurement model, it is obtained that the information from new measurements will be ignored. Thus, the result of the filter will become poor. The fading factor in Equation (9) is adopted to ensure the performance of the filter in the presence of dynamics model error. For improving the utilization rate of the new measurements, the fading factor $\alpha_{k}$ in Equation (8) becomes greater than 1. Subsequently, the variance matrix $P_{k|k-1}$ is inflated. Therefore, it is obtained that the contribution of ${\hat{x}}_{k|k-1}$ to ${\hat{x}}_{k|k}$ is reduced, and the impact of dynamics model error would be small.

Next, the fading factor $\alpha_{k}$ in Equation (5) is analyzed and derived via the innovation sequence orthogonal principle [28]

(19) $$E\{v_{k+j}v_{k}^{T}\}=0\;j=0,1,2,\ldots \;\;k=1,2,\ldots ,$$

where E{.} and $v_{k}$ are expected value and innovation sequence, respectively.

For the state model and measurement model stated by Equation (5), the lemma in [28] is given as follows:

**Lemma** **1.** *If $∥x_{k}-{\hat{x}}_{k|k}∥$ is significantly smaller than $∥x_{k}∥$, then, for any j,*

(20) $$\begin{array}{cc} C_{j,k} & =E\{v_{k+j}v_{k}\}\\ & =\Theta \left(k+j,\ldots ,k,{\hat{x}}_{k+j|k+j-1},\ldots ,{\hat{x}}_{k|k-1}\right)\left(P_{x_{k}y_{k}}-K_{k}C_{0,k}\right)\\ & =0, \end{array}$$

*where $P_{x_{k}y_{k}}$ is the cross covariance between state and measurement, $K_{k}$ represents the Kalman gain, $C_{0,k}$ equals to $E\{v_{k}v_{k}\}$, and $\Theta (k+j,\ldots ,k,{\hat{x}}_{k+j|k+j-1},\ldots ,{\hat{x}}_{k|k-1})$ can be written as follows:*

(21) $$\Theta \left(k+j,\ldots ,k,{\hat{x}}_{k+j|k+j-1},\ldots ,{\hat{x}}_{k|k-1}\right)=H_{k+j}\prod_{l=k+1}^{k+j-1}\Phi_{l}\left(I-K_{l}H_{l}\right)\Phi_{k}.$$

*The proof of Lemma 1 is omitted here. Please refer to Zhou et al. [28] if needed.*

It can be shown that Lemma 1 holds strictly for a linear system, and it is approximately true for a nonlinear system. A sufficient condition of Equation (20) is given as follows:

(22) $$P_{x_{k}y_{k}}-K_{k}C_{0,k}=0.$$

Using the common criterion of UKF leads to $K_{k}=P_{x_{k}y_{k}}P_{y_{k}y_{k}}^{-1}$, and

(23) $$P_{x_{k}y_{k}}\left(I-P_{y_{k}y_{k}}^{-1}C_{0,k}\right)=0,$$

where $P_{y_{k}y_{k}}$ is the error covariance matrix of measurement, and a sufficient condition of Equation (23) is given as follows:

(24) $$P_{y_{k}y_{k}}=C_{0,k}.$$

According to equations of KF that $P_{y_{k}y_{k}}=H_{k}P_{k|k-1}H_{k}^{T}+R_{k}$, Equation (24) can be rewritten as

(25) $$H_{k}P_{k|k-1}H_{k}^{T}+R_{k}-C_{0,k}=0.$$

If there exists a fading factor such that Equation (25) holds, the innovations $v_{k+j}$ and $v_{k}$ become approximately orthogonal. Thus, Equation (25) can be redescribed as

(26) $$H_{k}\alpha_{0}P_{k|k-1}H_{k}^{T}=C_{0,k}-R_{k}.$$

Noting that the terms $H_{k},P_{k},C_{0,k}$ and $R_{k}$ are full rank, by using the property of matrix trace, we have

(27) $$tr\left[H_{k}\alpha_{0}P_{k|k-1}H_{k}^{T}\right]=tr\left[C_{0,k}-R_{k}\right].$$

Because $\alpha_{0}$ is a scalar, then it can be expressed as follows:

(28) $$\alpha_{0}=\frac{tr[C_{0,k}-R_{k}]}{tr[H_{k}P_{k|k-1}H_{k}^{T}].}$$

It seems that Equation (28) can only be calculated if the state model (5) is a linear or linearized system. However, the linearization term $H_{k}P_{k|k-1}H_{k}^{T}$ can be readily approximated by the other nonlinear methods—for example, EKF and UKF, and so on. The focused UKF is utilized in this work. Therefore, the fading factor $\alpha_{0}$ can be applied to the UKF framework, and it is redescribed as follows:

(29) $$\alpha_{0}=\frac{tr[C_{0,k}-R_{k}]}{tr[P_{y_{k}y_{k}}-R_{k}]},$$

where $C_{0,k}$ can be expressed as follows:

(30) $$C_{0,k}=\left\{\begin{array}{cc} v_{k}v_{k}^{T}, & k=1,\\ \frac{\lambda C_{0,k-1}+v_{k}v_{k}^{T}}{1+\lambda }, & k>1, \end{array}\right.$$

where the forgetting factor λ is commonly set as 0.95 [19].

**Remark** **2.** *In the framework of the proposed filter, the fading factor $\alpha_{0}$ is a single fading factor. For the complicated multivariable systems in actual applications, it is not enough to use a fading factor. To overcome the shortcomings, Yang et al. [25] presented a multiple fading factor Kalman filter to deal with multivariable systems, and $\alpha_{0}$ is replaced by a matrix of fading factors $\Lambda =diag(\alpha_{0},\alpha_{1},\ldots ,\alpha_{n})$.*

## 3. The New Adaptive Robust Unscented Kalman Filter

In this section, we will focus on the proposed unscented Kalman filter based on fading factor and maximum correntropy criterion. The cost function of the proposed filter in Equation (15) has been derived, and it is embedded into the structure of UKF. In addition, it is well known that UKF has some good properties: easy implementation, appropriate performance and computational feasibility. Therefore, it is very popular in the nonlinear system.

Suppose that the nonlinear discrete-time system can be modelled as follows:

(31) $$x_{k}=f\left(x_{k-1}\right)+\omega_{k-1}\;\;\;y_{k}=h\left(x_{k}\right)+r_{k},$$

where $x_{k}$ is the state vector with the covariance matrix $P_{k}$, and the other terms have the same meaning as the above terms in Equation (5), respectively. The initial estimate state and its covariance matrix are given as ${\hat{x}}_{0}=E\left(x_{0}\right)$ and $P_{0}=E\left[\left(x_{0}-{\hat{x}}_{0}\right){\left(x_{0}-{\hat{x}}_{0}\right)}^{T}\right]$. Similarly, the estimate state and its covariance matrix at time k−1 are given as

(32) $${\hat{x}}_{k-1}=E\left(x_{k-1}\right),$$

(33) $$P_{k-1}=E\left[\left(x_{k-1}-{\hat{x}}_{k-1}\right){\left(x_{k-1}-{\hat{x}}_{k-1}\right)}^{T}\right].$$

The procedure of the proposed adaptive robust unscented Kalman filter depicted in Table 1 is described as follows:

Step 1: The Unscented Transform with 2*n* + 1 symmetric sigma points $\chi_{i,k-1}$ and weights update:

(34) $$\begin{array}{cc} \chi_{0,k-1} & ={\hat{x}}_{k-1},\\ \chi_{i,k-1} & ={\hat{x}}_{k-1}+\sqrt{n+\delta }{\left(\sqrt{P_{k-1}}\right)}_{i},\\ & \;i=1,2,\ldots ,n,\\ \chi_{i,k-1} & ={\hat{x}}_{k-1}-\sqrt{n+\delta }{\left(\sqrt{P_{k-1}}\right)}_{i},\\ & \;i=n+1,n+2,\ldots ,2n, \end{array}$$

where $\delta =\varphi^{2}\left(n+\kappa \right)-n$, φ, which ranges from 0 to 1, controls the distribution of sigma points. κ equals 3−n, and $\sqrt{P_{k-1}}$ is the Cholesky factor of $P_{k-1}$, respectively.

The corresponding weights $\omega_{0}^{m}$ and covariance $\omega_{0}^{c}$ are as follows:

(35) $$\begin{array}{cc} \omega_{0}^{m} & =\frac{\delta }{n+\delta },\\ \omega_{0}^{c} & =\frac{\delta }{n+\delta }+\left(1-\varphi^{2}+\eta \right),\\ \omega_{i}^{m} & =\omega_{i}^{c}=\frac{1}{2(n+\delta )}\;\;\;\;\;\;\;i=1\ldots .,2n, \end{array}$$

where η is set as 2 generally for Gaussian distribution and relates with the prior distribution of state.

Step 2: Time update

(36) $$\chi_{i,k|k-1}=f\left(\chi_{i,k-1}\right)\;{\hat{x}}_{k|k-1}=\sum_{i=0}^{2n}\omega_{i}^{m}\chi_{i,k|k-1}.$$

Step 3: Modified measurement covariance update

(37) $$y_{i,k|k-1}=h\left(\chi_{i,k-1}\right)\;{\hat{y}}_{k|k-1}=\sum_{i=0}^{2n}\omega_{i}^{m}y_{i,k|k-1},$$

(38) $$\xi_{k}=R_{k}^{-1/2}\left({\hat{y}}_{k|k-1}-y_{k}\right),$$

(39) $$\Psi =\mathrm{diag}[\psi_{k,j}\left(\xi_{k,j}\right)]\;j=1,2,\ldots ,m,$$

(40) $${\tilde{R}}_{k}=R_{k}^{T/2}\Psi^{-1}R_{k}^{1/2}.$$

Step 4: Fading factor update

(41) $$v_{k}=y_{k}-{\hat{y}}_{k|k-1},$$

(42) $$C_{0,k}=\left\{\begin{array}{cc} v_{k}v_{k}^{T}, & k=1,\\ \frac{\lambda C_{0,k-1}+v_{k}v_{k}^{T}}{1+\lambda }, & k>1, \end{array}\right.$$

(43) $$\alpha_{0}=\frac{tr[C_{0,k}-R_{k}]}{tr\left[\sum_{i=0}^{2n}\omega_{i}^{c}\left(y_{k,i}-{\hat{y}}_{k|k-1}\right){\left(y_{k}-{\hat{y}}_{k|k-1}\right)}^{T}\right]},$$

(44) $$\alpha_{k}=\left\{\begin{array}{cc} \alpha_{0}, & \alpha_{0}>1,\\ 1, & \alpha_{0}\leq 1. \end{array}\right.$$

Step 5: Measurement update

(45) $$P_{k|k-1}=\alpha_{k}\left[\sum_{i=0}^{2n}\omega_{i}^{c}\left(\chi_{i,k|k-1}-{\hat{x}}_{k|k-1}\right){\left(\chi_{i,k|k-1}-{\hat{x}}_{k|k-1}\right)}^{T}+Q_{k}\right],$$

(46) $$P_{y_{k}y_{k}}=\alpha_{k}\sum_{i=0}^{2n}\omega_{i}^{c}\left(y_{k,i}-{\hat{y}}_{k|k-1}\right){\left(y_{k,i}-{\hat{y}}_{k|k-1}\right)}^{T}+{\tilde{R}}_{k},$$

(47) $$P_{x_{k}y_{k}}=\alpha_{k}\sum_{i=0}^{2n}\omega_{i}^{c}\left(\chi_{i,k|k-1}-{\hat{x}}_{k|k-1}\right){\left(y_{i,k|k-1}-{\hat{y}}_{k|k-1}\right)}^{T},$$

(48) $$P_{k|k}=P_{k|k-1}-K_{k}P_{y_{k}y_{k}}K_{k}^{T},$$

(49) $$K_{k}=P_{x_{k}y_{k}}P_{y_{k}y_{k}}^{-1},$$

(50) $${\hat{x}}_{k|k}={\hat{x}}_{k|k-1}+K_{k}\left(y_{k}-{\hat{y}}_{k|k-1}\right).$$

**Remark** **3.** *In the process of nonlinear filter, it can be seen that both fading factor and maximum correntropy criterion are applied in the cost function of the new filter. We can obtain that the estimate state ${\hat{x}}_{k}$ is more accurate than estimate state ${\hat{x}}_{k}$ using other nonlinear filters such as EKF, UKF and other common nonlinear filters, etc. First, the proposed filter has better adaptive ability to balance the contribution between the process model information and measurement on the state vector. Second, the proposed filter can retain the effect of outliers. Particularly, when the state or/and measurement model are contaminated by outliers, the effectiveness of the proposed filter is much better than that of UKF.*

## 4. Simulation and Comparison

To illustrate the practical applicability of the proposed nonlinear filtering algorithm, two classical filtering applications are employed in this section.

### 4.1. Radar Tracking System

We track a moving object by a radar which utilizes measurements of distances information. The two-dimensional system uses a single station for tracking targets, and the state vector includes position and velocity. The dynamics model moves within two dimensional plane according to the standard dynamics model [29]

(51) $$X_{k}=\Phi X_{k-1}+\Gamma u_{k},$$

where $X_{k}=\left[x,\dot{x},y,\dot{y}\right]$, *x* and *y* are Cartesian coordinates of the moving target, and $\dot{x}$ and $\dot{y}$ are correlative velocities of the moving target, respectively. $u_{k}$ is zero mean Gaussian white noise with the covariance $Q=diag(\left[10^{-4},10^{-4}\right])$. The transition matrix and noise excitation matrix are as follows:

(52) $$\Phi =\left[\begin{array}{cccc} 1 & T & 0 & 0\\ 0 & 1 & 0 & 0\\ 0 & 0 & 1 & T\\ 0 & 0 & 0 & 1 \end{array}\right]\;\mathrm{and}\;\;\Gamma =\left[\begin{array}{cc} T^{2}/2 & 0\\ T & 0\\ 0 & T^{2}/2\\ 0 & T \end{array}\right],$$

where T=1 is the time interval, and the measurement equation is

(53) $$Z_{k}=\sqrt{{\left(x_{k}-o_{x}\right)}^{2}+{\left(k_{k}-o_{y}\right)}^{2}}+v_{k},$$

where $\left(o_{x},o_{y}\right)$ = (0, 1000) is land station, the variance of $r_{k}$ is R=5, and $v_{k}$ is tuning parameter satisfying v≪1.

Next, we will analyze and compare estimation performance of the following nonlinear filters that are conventional unscented Kalman filter (UKF), adaptive Huber unscented Kalman filter (AHUKF) [24], and the proposed filtering algorithm (AMUKF). Those three filters are utilized to track position and velocity of the maneuvering vehicle.

The initial position and velocity of the maneuvering vehicle are set as [0 m, 1400 m] and [2 m/s, −10 m/s], respectively. The value of initial variance $P_{0}$ is diag[1,1,1,1]. Simulation time lasts 50 s, the parameter σ of Gaussian kernel function is set as 0.8 here. In this simulation, two cases are considered: (1) measurement outliers and (2) state and measurement outliers simultaneously.

**Figure 1:**
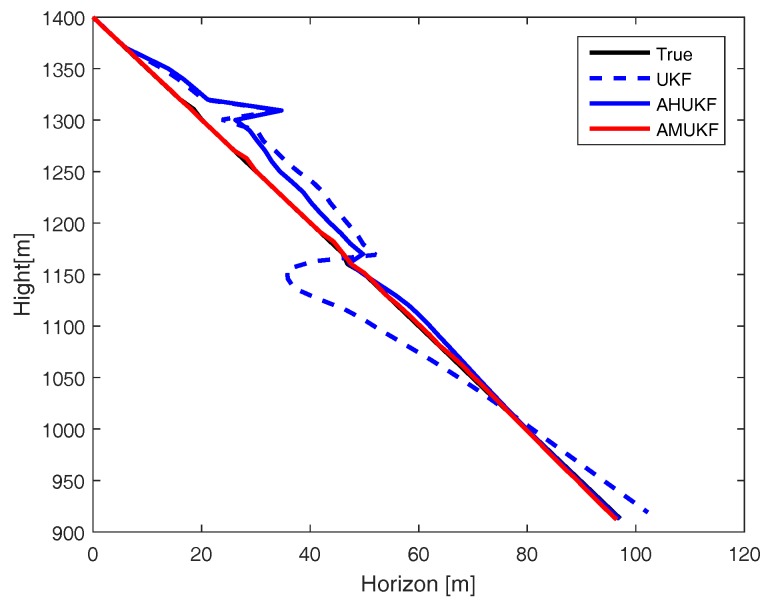
Target tracking performance in case 1.

**Figure 2:**
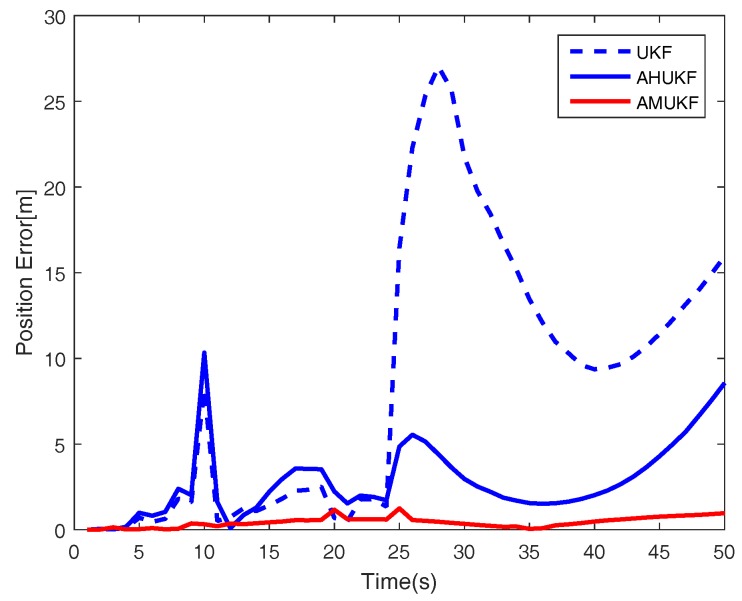
Position tracking performance in case 1.

**Figure 3:**
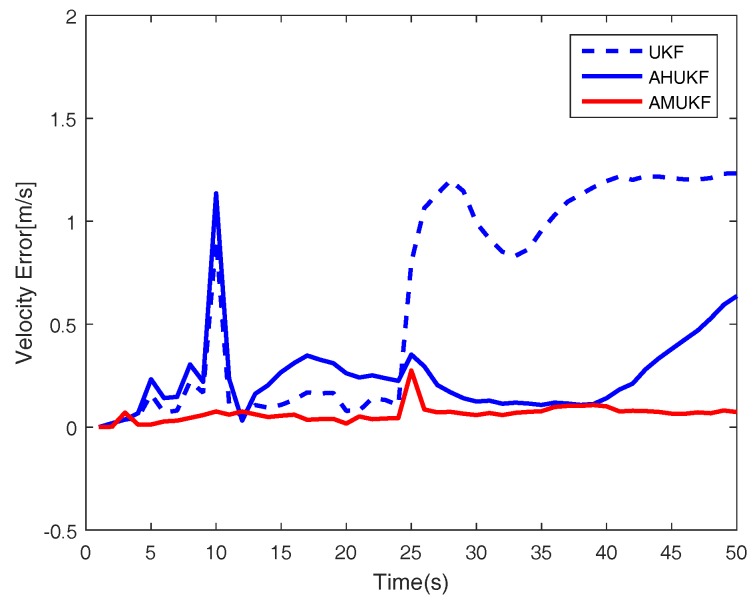
Velocity tracking performance in case 1.

For the first case, we set the measurement outliers ${\hat{Z}}_{20}=Z_{20}+2$ (m) and ${\hat{Z}}_{35}=Z_{35}-1$ (m). Under the same condition, Figure 1, Figure 2 and Figure 3 show the tracking performance of UKF, AHUKF and the proposed filter (AMUKF). Figure 1 shows the tracking results of the three filters above, and the effects of AMUKF are better than that of other filters. The performance of AHUKF and AMUKF is much better than that of UKF because the UKF is a nonlinear extension of the KF, and it is susceptible to outliers. In Figure 1, we can see that AHUKF has a robust property to some extent, and it can be seen that the impact of state outlier is to be suppressed to a certain extent. However, comparing with AMUKF, it is not better than that of AMUKF. In summary, we can draw the conclusion that AMUKF can suppress the outliers and has better robust properties. Thus, it has a better tracking result for true state. The tracking errors obtained by AHUKF are bigger than that obtained by AMUKF. Figure 2 and Figure 3 show the position errors and velocity errors about the three filters, respectively. In contrast, under the measurement outlier, the estimation position and velocity errors for AMUKF are always smaller than that of other filters.

**Figure 4:**
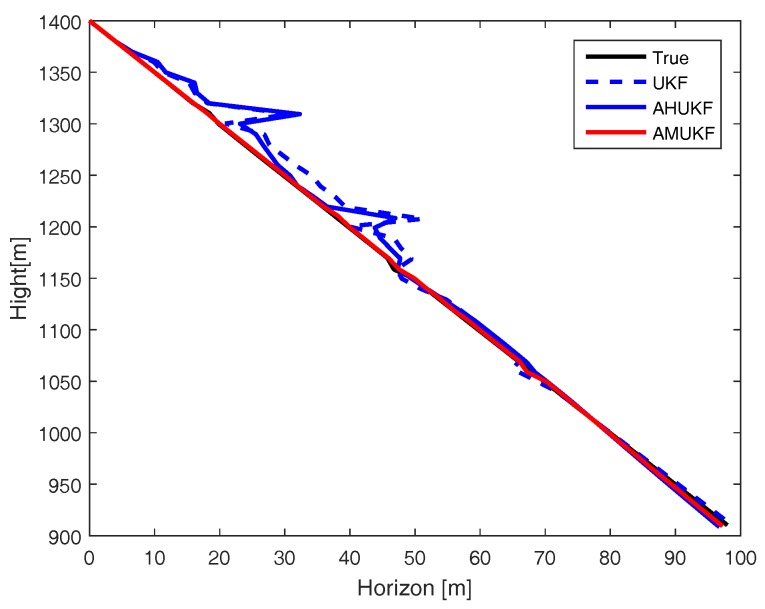
Target tracking performance in case 2.

**Figure 5:**
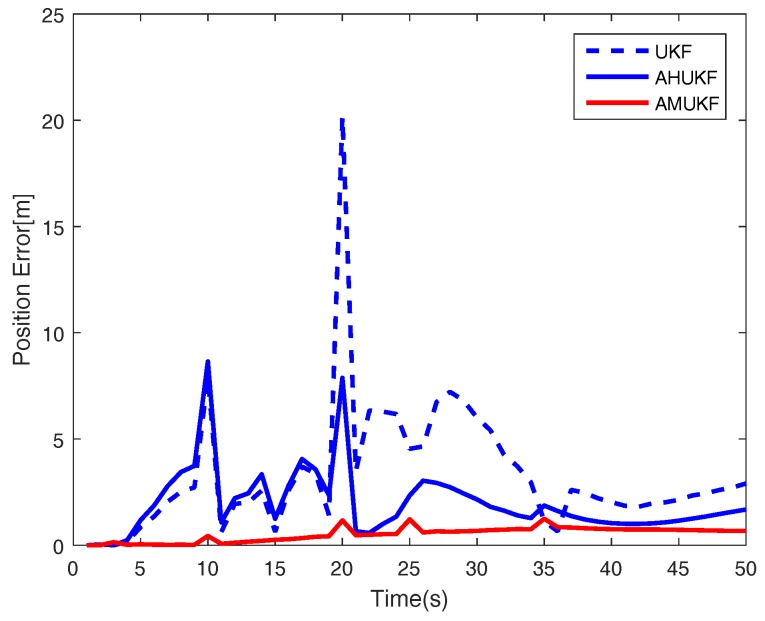
Position tracking performance in case 2.

**Figure 6:**
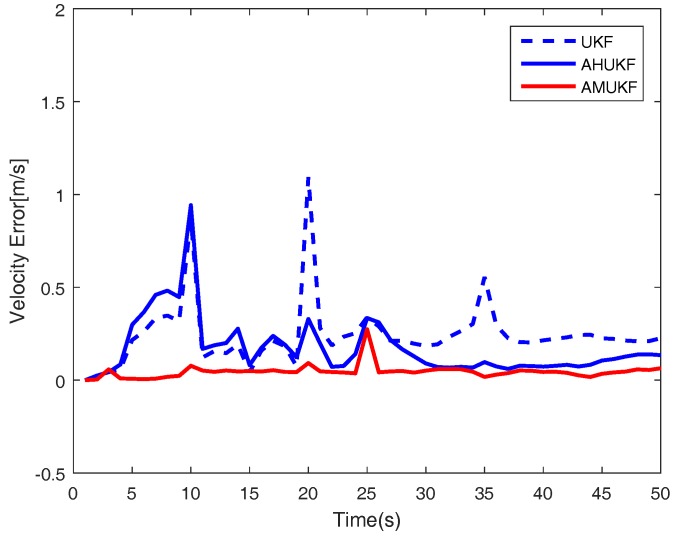
Velocity tracking performance in case 2.

For the second case, we also estimate position and velocity like the first case. The state outliers are set as ${\hat{X}}_{10}=X_{10}$ + 0.2 × [5,1,5,1]${}^{T}$ (m), ${\hat{X}}_{25}=X_{25}$ − 0.4 × [5,1,5,1] ${}^{T}$ (m), and the measurement outliers are set as ${\hat{Z}}_{20}=Z_{20}+2$ (m)$,{\hat{Z}}_{35}=Z_{35}-5$ (m), respectively. From Figure 4, Figure 5 and Figure 6, we can obtain that the effect of the three different filters when the process and measurement have outliers simultaneously. From Figure 4, it can be seen that the tracking results of the three filters in case 2, and the performance of AMUKF is better than that of other filters. The results of AHUKF and AMUKF are much better than that of UKF because AHUKF has robust properties, the effectiveness of AHUKF is approaching that of AMUKF, but it is not better than that of AMUKF. The tracking errors obtained by AHUKF are bigger than that obtained by AMUKF. Figure 5 and Figure 6 show the position errors and velocity errors about the three filters, respectively. Finally, it can be concluded that the errors of AMUKF are smaller than that of AHUKF. From Figure 1 and Figure 4, we also see that the behaviours of three filters are convergent. Finally, the root mean square error (RMSE) of state is shown in Table 2, which shows that the presented filtering algorithm outperforms both UKF and AHUKF.

### 4.2. Mars Entry Model

In this subsection, the Mars entry model is considered. During Mars entry, the vehicle is affected by some uncertainty disturbances such as lift, drag, gravity, and measurement outliers, etc. Measurement outliers are investigated. The dynamics of the entry vehicle are described by [30]

(54) $$\begin{array}{c} \left[\begin{array}{c} \dot{r}\\ \dot{v} \end{array}\right]=\left[\begin{array}{c} v\\ -D\frac{v}{∥v∥}-L\cos\tilde{\sigma }\frac{v}{∥v∥}\times \frac{v\times r}{∥v\times r∥}+L\sin\tilde{\sigma }\frac{v\times r}{∥v\times r∥}-g\frac{r}{∥r∥} \end{array}\right], \end{array}$$

where $r={\left[r_{x},r_{y},r_{z}\right]}^{T}$ and $v={\left[v_{x},v_{y},v_{z}\right]}^{T}$ denote position vector and velocity vector, and $\tilde{\sigma }$ is bank angle. The lift acceleration *L*, drag acceleration *D* and gravitational acceleration *g* are

(55) $$L=\frac{1}{2}\tilde{\rho }{∥v∥}^{2}s/C_{L}M\;\;\;\;D=\frac{1}{2}\tilde{\rho }{∥v∥}^{2}s/C_{D}M\;\;\;\;g=\frac{\tilde{g}}{R^{2}},$$

where ∥r∥ and ∥v∥ are the scalar values of r and v, *M* is the mass of the vehicle, and $\tilde{g}$ is the planet’s gravitational constant. Atmosphere density $\tilde{\rho }$ is given by

(56) $$\begin{array}{c} \tilde{\rho }={\tilde{\rho }}_{0}\exp\left(\frac{r_{0}-∥r∥}{h_{s}}\right), \end{array}$$

where ${\tilde{\rho }}_{0}=2\times 10^{-4}$ kg/m${}^{3}$ is the nominal reference density, $r_{0}=3.4372\times 10^{6}$ m is the reference radius of Mars (40 km above Mars surface), and $h_{s}=7500$ m is the constant scale height.

For the measurement model, the two Mars orbiters–Mars Reconnaissance Orbiter (MRO) and Mars Express (MEX) used by Curiosity for relay communications are employed. The movement of the orbiter in polar coordinate can be constructed [31]:

(57) $$\begin{array}{c} r_{ob}=\frac{a(1-e^{2})}{1+ecos(\theta -\bar{\omega })}\;\;\;\;r_{ob}^{2}\dot{\theta }=\sqrt{\tilde{g}a\left(1-e^{2}\right)}, \end{array}$$

where $r_{ob}$ is the distance between the orbiter and the center of Mars, θ is the angle between the ascending node and the orbiter, *a* is the semi-major axis of the movement ellipse, *e* is the eccentricity ratio, $\bar{\omega }$ is the argument of perigee, and $\tilde{g}$ is the gravity constant of Mars. The ecliptic longitude and latitude of the orbiter are:

(58) $$\Omega_{ob}=\left\{\begin{array}{cc} -\cos^{-1}\left[\frac{\cos\theta }{\cos\phi_{ob}}\right]+\Omega , & \phi_{ob}\leq 0,\\ \cos^{-1}\left[\frac{\cos\theta }{\cos\phi_{ob}}\right]+\Omega , & \phi_{ob}>0, \end{array}\right.$$

where $\phi_{ob}=\sin^{-1}\left[\frac{\sin\theta \sin i}{\sin k}\right]$, $\Omega_{ob}$ is ecliptic longitude, and $\phi_{ob}$ is the ecliptic latitude, and *i* is orbital inclination (angle between longitude and latitude, $k=\frac{\pi }{2}$). Thus, the relative range measurement between the vehicle and the orbiter can be constructed:

(59) $$\begin{array}{c} \begin{array}{c} R_{ob}=\sqrt{{\left(r-r_{ob}\right)}^{T}\left(r-r_{ob}\right)},\\ {\tilde{R}}_{ob}=R_{ob}+\varepsilon_{ob}, \end{array} \end{array}$$

where $r_{ob}={\left(\cos\phi_{ob}\cos\Omega_{ob}\;\;\cos\phi_{ob}\sin\Omega_{ob}\;\;\sin\phi_{ob}\right)}^{T}$, and $\varepsilon_{ob}$ is the range measurement noise, whose prior covariance is set to be 100, meaning the range measurement error is 10 m.

The relative range measurements between Mars surface beacons (MSBs) and the vehicle can be obtained by:

(60) $$\begin{array}{c} \begin{array}{c} {\tilde{R}}_{B}^{\hat{i}}=R_{B}^{\hat{i}}+\varepsilon_{R}^{\hat{i}}, \end{array} \end{array}$$

where $R_{B}^{\hat{i}}=\sqrt{{\left(x_{B}^{\hat{i}}-r_{x}\right)}^{2}+{\left(y_{B}^{\hat{i}}-r_{y}\right)}^{2}+{\left(z_{B}^{\hat{i}}-r_{z}\right)}^{2}}$ refers to the true range from the entry vehicle to the *i*th beacon, $\tilde{R_{B}}$ is the measurement range, and $x_{B}^{\hat{i}},y_{B}^{\hat{i}}$ and $z_{B}^{\hat{i}}$ are the position coordinates of MSBs. $\varepsilon_{R}^{\hat{i}}$ is the range measurement noise.

The observation of rate measurement can be given as:

(61) $$\begin{array}{c} \begin{array}{c} {\tilde{V}}_{B}^{\hat{i}}=V_{\hat{i}}+\varepsilon_{V}^{\hat{i}}\;\;\;V_{B}^{\hat{i}}=\frac{dR_{\hat{i}}}{dt}\;\;\;\hat{i}=1,\cdots ,N, \end{array} \end{array}$$

where $V_{B}^{\hat{i}}$ is the true rate, ${\tilde{V}}_{B}^{\hat{i}}$ is the measured rate, and $\varepsilon_{V}^{\hat{i}}$ is the rate measurement noise, whose prior covariance is set to be 1, meaning that the measurement error rate is 1 m/s.

From the above measurement models, the overall observation model can be obtained as

$$y=\left(\begin{array}{c} {\tilde{R}}_{ob}\\ {\tilde{R}}_{B}\\ {\tilde{V}}_{B} \end{array}\right).$$

In this simulation, the measurement outliers are considered. We utilize UKF and AHUKF [24] to compare with the proposed filtering algorithm too. Their performance is to be analyzed as follows. First, the initial values are listed in Table 3. The parameters of the two orbiters are listed in Table 4. The simulation time is 500 s.

**Figure 7:**
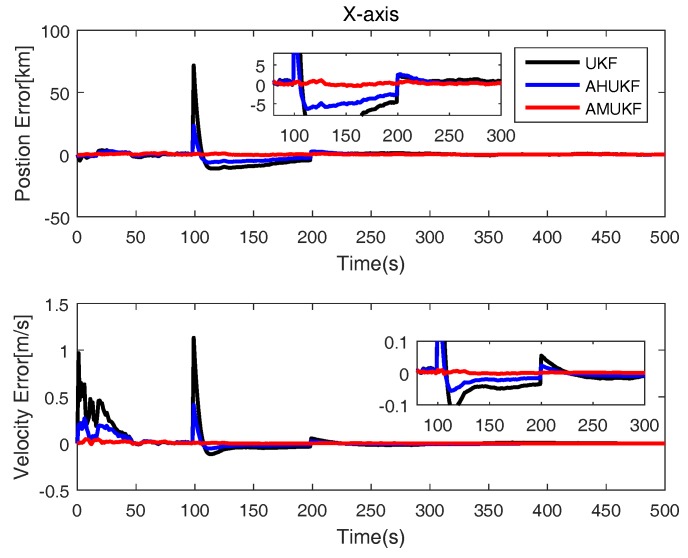
Position and velocity tracking errors on the *x*-axis.

**Figure 8:**
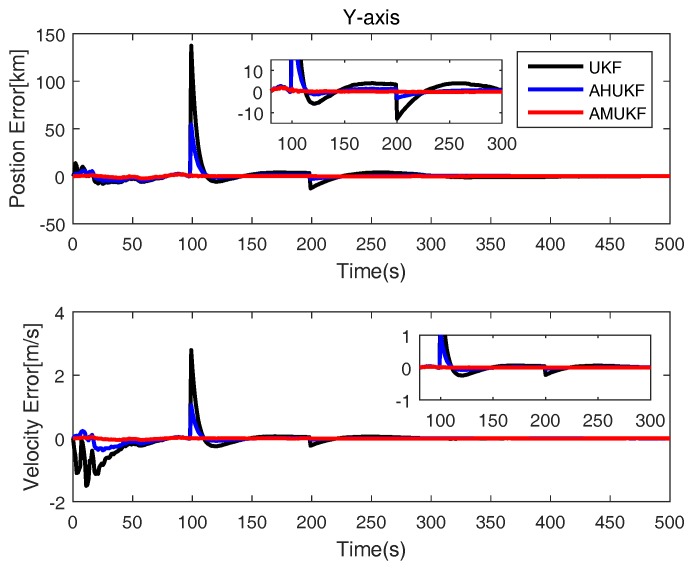
Position and velocity tracking errors on the *y*-axis.

**Figure 9:**
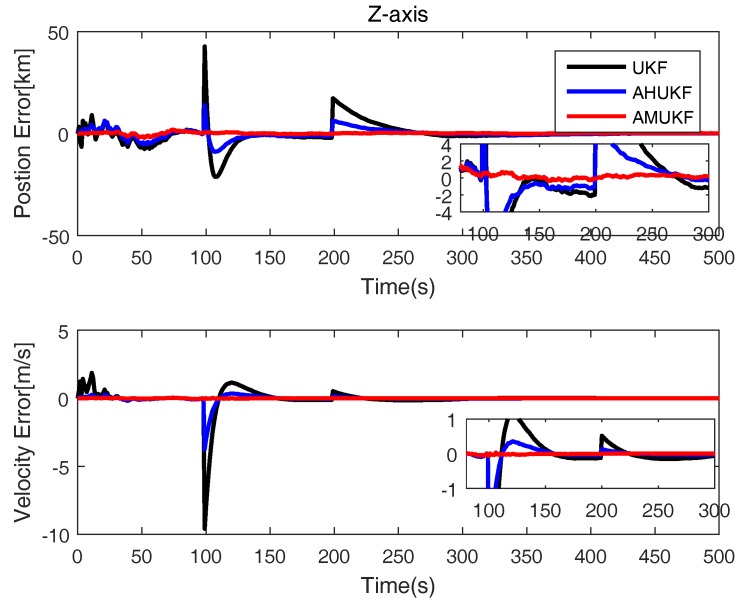
Position and velocity tracking errors on the *z*-axis.

The initial position and velocity of maneuvering vehicle are also showed in Table 3. The initial errors matrix $P_{0}=diag\left(\left[1000;1000;1000;1000;10;10\right]\right)$. $Q=10^{-5}\times diag\left(\left[0.1;0.1;0.1;0.1;0.1;0.1\right]\right)$; R=diag([100;100;100;1;1;1]). The parameter σ of Gaussian kernel function is set as 10 here. The measurement outliers are assumed as ${\tilde{y}}_{100}=y_{100}$ + 0.3 $\times {\left[800,800,800,800,30,30\right]}^{T}$ and ${\tilde{y}}_{200}=y_{200}-0.4*{\left[800,800,800,800,30,30\right]}^{T}$, respectively. From Figure 7, Figure 8 and Figure 9, obviously, it can be seen that the state errors of UKF become very large for measurement outliers. while others’ errors are very small. Therefore, we can determine that the performance of AHUKF and AMUKF is far better than that of UKF, especially AMUKF. Even when the measurement outliers occur, AMUKF can effectively track the movement of the vehicle. Finally, from Figure 7, Figure 8 and Figure 9, we also see that the behaviours of three filters are convergent.

## 5. Conclusions

In this paper, a new adaptive robust unscented Kalman filter is obtained by combining both the robust property of maximum correntropy criterion and the adaptive property of the fading factor with the superiority of UKF. The cost function of the proposed filter includes both the fading factor to the state model and the maximum correntropy criterion to the measurement model. The maximum correntropy criterion can enhance the ability of being insensitive to outliers, and the fading factor can improve the ability of strongly tracking the state estimate. Therefore, the proposed filtering algorithm can track the state strongly and is insensitive to outliers. After that, the process of fading factor is derived in detail. Two numerical examples are given to illustrate the effectiveness of the proposed filter. UKF and AHUKF are selected for comparison with the proposed filtering algorithm in simulation. When state or/and measurement outliers occur, the proposed filter can track the target effectively and suppress the effect of outliers. Therefore, from the theoretical and numerical simulation results, it can be concluded that the proposed filter has not only the robustness of the fading factor but also the accuracy and flexibility of nonlinear systems. In the future, we will study the stability of the adaptive robust unscented Kalman filter based on the maximum correntropy criterion.

## Figures and Tables

**Table 1:** AMUKF. AMUKF is short for Adaptive Robust Unscented Kalman Filter.

First set $x_{0,k|k}$ = [0 m, 1400 m, 2 m/s, −10 m/s]${}^{T}$.
$P_{0,k|k}=diag\left[1,1,1,1\right]$.
Then iterate the follow,
for i=0:N−1
Reformulate the augmented covariance
time update
$\chi_{i,k|k-1}=f\left(\chi_{i,k-1}\right)$
${\hat{x}}_{k|k-1}=\sum_{i=0}^{2n}\omega_{i}^{m}\chi_{i,k|k-1}$
Measurement update:
${\hat{x}}_{i,k|k-1}=\left[\chi_{i,k|k-1}\;\;\chi_{i,k|k-1}+\mu \left(\sqrt{P_{i,k|k-1}}\right)\;\;\chi_{i,k|k-1}-\mu \left(\sqrt{P_{i,k|k-1}}\right)\right]$
$y_{i,k|k-1}=h\left({\hat{x}}_{i,k|k-1}\right)$
${\hat{y}}_{k|k-1}=\Sigma_{i=0}^{2n}\omega_{k}y_{i,k|k-1}$
Calculate $\alpha_{k}$
$v_{k}=y_{k}-{\hat{y}}_{k|k-1}$
$C_{0,k}=\left\{\begin{array}{cc} v_{k}v_{k}^{T} & k=1\\ \frac{\lambda C_{0,k-1}+v_{k}v_{k}^{T}}{1+\lambda } & k>1 \end{array}\right.$
$\alpha_{k}=\left\{\begin{array}{cc} \alpha_{0} & \alpha_{0}>1\\ 1 & \alpha_{0}\leq 1 \end{array}\right.$
$P_{k|k-1}=\alpha_{k}\mathrm{tr}\left[\sum_{i=0}^{2n}\omega_{i}^{c}\left(E_{i}\right){\left(E_{i}\right)}^{T}+Q_{k}\right]$
$P_{y_{k}y_{k}}=\alpha_{k}\sum_{i=0}^{2n}\omega_{i}^{c}\left(y_{i,k|k-1}-{\hat{y}}_{k|k-1}\right){\left(y_{i,k|k-1}-{\hat{y}}_{k|k-1}\right)}^{T}+{\tilde{R}}_{k}$
$P_{x_{k}y_{k}}=\alpha_{k}\sum_{i=0}^{2n}\omega_{i}^{c}\left(\chi_{i,k|k-1}-{\hat{y}}_{k|k-1}\right){\left(y_{i,k|k-1}-{\hat{y}}_{k|k-1}\right)}^{T}$
$K_{k}=P_{x_{k}y_{k}}P_{y_{k}y_{k}}^{-1}$ ${\hat{x}}_{k|k}={\hat{x}}_{k|k-1}+K_{k}\left(y_{k}-{\hat{y}}_{k|k-1}\right)$
$P_{k|k}=P_{k|k-1}-K_{k}P_{y_{k}y_{k}}K_{k}^{T}$
end
where $E_{i}=\chi_{i,k|k-1}-{\hat{x}}_{k|k-1}$, $\mu =\sqrt{n+\kappa }$,
$W_{0}=\kappa /\left(n+\kappa \right)$, $W_{i}=\kappa /\left(n+\kappa \right),$
i=1,2,⋯,2n and κ is a tune parameter.
More details about the selection of κ can be seen in [11,22].

**Table 2:** RMSE of State. RMSE is short for root mean square error.

Filter	RMSE of *x*	RMSE of $\dot{x}$	RMSE of *y*	RMSE of $\dot{y}$
UKF	28.1	0.315	145	0.117
AHUKF	26.7	0.017	139	0.037
AMUKF	25.8	0.011	135	0.029

**Table 3:** Parameters setting of initial conditions. MSBs is short for Mars surface beacons.

Initial Setting	Notation	Values
Initial position	$\left(r_{x_{0}},r_{y_{0}},r_{z_{0}}\right)$	(−3.92 km, −3099.09 km, −1663.11 km)
Initial velocity	$\left(v_{x_{0}},v_{y_{0}},v_{z_{0}}\right)$	(463.25 m/s, −1528.75 m/s, 5268.14 m/s)
MSBs’ locations (1)	$\left(x_{B}^{1},y_{B}^{1},z_{B}^{1}\right)$	(875.35 km, −2914.43 km, −1509.77 km)
MSBs’ locations (2)	$\left(x_{B}^{2},y_{B}^{2},z_{B}^{2}\right)$	(410.25 km, −2955.32 km, −1624.04 km)
Vehicle mass	M	2804 kg
Vehicle cross-section	s	15.9 m${}^{2}$

**Table 4:** Parameters of the orbiters. MRO and MEX are short for Mars Reconnaissance Orbiter and Mars Express, respectively.

	MRO	MEX
semi-major axis *a*	3663.7 km	8572.2 km
eccentricity ratio *e*	0.0089 rad	0.5770 rad
argument of perigee $\bar{\omega }$	4.7124 rad	2.7911 rad
orbital inclination *i*	1.6154 rad	1.5055 rad
